# Resistance to cardiomyocyte hypertrophy in *ae3*^*−/−*^ mice, deficient in the AE3 Cl^−^/HCO_3_^−^ exchanger

**DOI:** 10.1186/1471-2261-14-89

**Published:** 2014-07-21

**Authors:** Daniel Sowah, Brittany F Brown, Anita Quon, Bernardo V Alvarez, Joseph R Casey

**Affiliations:** 1 Department of Biochemistry and Membrane Protein Disease Research Group, University of Alberta, Edmonton T6G 2H7, Canada; 2 Centro de Investigaciones Cardiovasculares, Facultad de Ciencias Medicas, Universidad Nacional de La Plata, La Plata, Argentina

**Keywords:** AE3, Bicarbonate transport, Chloride/bicarbonate exchange, pH regulation, Cardiomyocyte hypertrophy, Heart failure

## Abstract

**Background:**

Cardiac hypertrophy is central to the etiology of heart failure. Understanding the molecular pathways promoting cardiac hypertrophy may identify new targets for therapeutic intervention. Sodium-proton exchanger (NHE1) activity and expression levels in the heart are elevated in many models of hypertrophy through protein kinase C (PKC)/MAPK/ERK/p90^RSK^ pathway stimulation. Sustained NHE1 activity, however, requires an acid-loading pathway. Evidence suggests that the Cl^−^/HCO_3_^−^ exchanger, AE3, provides this acid load. Here we explored the role of AE3 in the hypertrophic growth cascade of cardiomyocytes.

**Methods:**

AE3-deficient (*ae3*^*−/−*^) mice were compared to wildtype (WT) littermates to examine the role of AE3 protein in the development of cardiomyocyte hypertrophy. Mouse hearts were assessed by echocardiography. As well, responses of cultured cardiomyocytes to hypertrophic stimuli were measured. pH regulation capacity of *ae3*^*−/−*^ and *WT* cardiomyocytes was assessed in cultured cells loaded with the pH-sensitive dye, BCECF-AM.

**Results:**

*ae3*^*−/−*^ mice were indistinguishable from wild type (WT) mice in terms of cardiovascular performance. Stimulation of *ae3*^*−/−*^ cardiomyocytes with hypertrophic agonists did not increase cardiac growth or reactivate the fetal gene program. *ae3*^*−/−*^ mice are thus protected from pro-hypertrophic stimulation. Steady state intracellular pH (pH_i_) in *ae3*^*−/−*^ cardiomyocytes was not significantly different from WT, but the rate of recovery of pH_i_ from imposed alkalosis was significantly slower in *ae3*^*−/−*^ cardiomyocytes.

**Conclusions:**

These data reveal the importance of AE3-mediated Cl^−^/HCO_3_^−^ exchange in cardiovascular pH regulation and the development of cardiomyocyte hypertrophy. Pharmacological antagonism of AE3 is an attractive approach in the treatment of cardiac hypertrophy.

## Background

Cardiovascular diseases remain a major cause of death worldwide despite progress in disease outcomes of patients [1]. Heart failure (HF) is the common end-stage of many cardiovascular disorders, with a prevalence of 5.8 million in the USA and about 23 million worldwide [2,3]. Annually, 550,000 new cases of HF arise in the U.S.A. The intricate molecular events resulting in heart failure remain incompletely understood, but enlargement of cardiac contractile cells (cardiomyocyte hypertrophy) in response to various stimuli is central to the progression to heart failure [4].

Cardiac cells are terminally differentiated cells that respond to increased stress by increasing their size rather than mitotically dividing to increase their number [5]. Cardiovascular events that increase myocardial stress (workload) chronically induce hypertrophic growth. Pressure overload, myocardial infarction, obesity, pregnancy or exercise can independently trigger molecular mechanisms culminating in increased cardiomyocyte size. Cardiac hypertrophy occurs to normalize the elevated demand on the myocardium, and can be physiological or pathological depending on the source of the initiating stimuli [6]. Physiological hypertrophy prevails in healthy individuals during increased physical activities or in pregnant women. Pathological hypertrophy results from prolonged elevated blood pressure (pressure overload), ischemia accompanied by changes in Ca^++^ handling, or genetic abnormalities. Initially, pathological hypertrophic growth compensates for the decline in contractile function, but ultimately the myocardium becomes decompensated from sustained exposure to the initiating stimuli. Understanding the distinct pathways mediating cardiac hypertrophic development has potential to identify new drug targets for the management of heart failure.

Intracellular pH (pH_i_) regulation is paramount in maintaining normal cardiac function [7,8]. Plasma membrane transporters involved in maintaining pH_i_ at physiological levels in the heart include the Na^+^/H^+^ exchanger (NHE1), Na^+^/HCO_3_^−^ co-transporters (NBC), and Cl^−^/HCO_3_^−^ exchangers [9,10]. Cytosolic acidification or hormonal stimulation activate NHE1, which facilitates electroneutral Na^+^/H^+^ exchange, to alkalinize the cytosol [11]. Accumulating evidence suggests that NHE1 expression level and activity increase in hypertrophy [12,13]. In the hypertrophied myocardium of the spontaneously hypertensive rats (SHR), there was an increased activation of NHE1 [14] and NHE1 inhibition reduced cardiac hypertrophy and interstitial fibrosis [15]. Transgenic mice expressing activated NHE1 exchanger had enlargement of the heart and increased sensitivity to hypertrophic stimulation [16]. Since NHE1 activation induces acid extrusion, alkalinization should accompany NHE1 activation. NHE1 activation was not, however, accompanied by increased pH_i_, although cytosolic Na^+^ was elevated [14]. Moreover, under alkaline conditions, NHE1 activity is self-inhibited, which suggests that an acidifying mechanism running counter to NHE1 is necessary for sustained NHE1 activation [17-20]. Indeed, Cl^−^/HCO_3_^−^ exchange mediated by AE3 provides this acidifying pathway [7,8,10].

The heart expresses three Cl^−^/HCO_3_^−^ exchanger isoforms: AE1, AE2 and AE3 [10,21]. Another cardiac Cl^−^/HCO_3_^−^, SLC26a6, [22-24], may represent the Cl^−^/OH^−^ exchanger (CHE) that has been reported in the heart [25]. Two AE3 variants, AE3 full length (AE3fl) and cardiac AE3 (AE3c) are expressed in the heart; AE3fl is also expressed in the brain and retina [26-28]. Phenylephrine (PE) and angiotensin II (ANGII), acting on their G-protein-coupled receptors (GPCRs), activate AE3fl via protein kinase C (PKC). Interestingly, PKC can indirectly activate NHE1 via MAPK-dependent mechanisms [29]. Moreover, carbonic anhydrase II (CAII), another modulator of the PE-dependent hypertrophic growth, interacts with both NHE1 and AE3 to provide their respective transport substrates, H^+^ and HCO_3_^−^[30,31].

CAII activation was recently found to be important in the induction of cardiomyocyte hypertrophy. In isolated rat cardiomyocytes, inhibition of CAII catalytic activity reduced phenylephrine (PE) and angiotensin II (ANGII) induced cardiomyocyte hypertrophy [32]. Additionally, infection of neonatal rat cardiomyocytes with adenoviral constructs encoding catalytically inactive CAII mutant, CAII-V143Y, reduced the response of the cardiomyocytes to hypertrophic stimuli, suggested to arise from a dominant negative mode of action [33]. Cardiomyocytes from CAII-deficient mice had physiological hypertrophy, but were unresponsive to hypertrophic stimulation [33]. Finally, expression of CAII and CAIV was elevated in the hypertrophic ventricles from failing human hearts, indicating that elevation of carbonic anhydrases is a feature of heart failure in people [34]. Taken together, these findings show that CAII plays a role in the development of cardiomyocyte hypertrophy.

Several reports revealed that CAII physically and functionally interacts with Cl^−^/HCO_3_^−^ anion exchangers to enhance the transport activity of anion exchangers forming a bicarbonate transport metabolon [31,35-37], although some reports have questioned the physiological relevance of this physical and functional linkage [38-40]. CAII also interacts physically and functionally with NHE1 to increase the exchange activity [30,41]. These observations suggest that simultaneous activation of AE3, CAII and NHE1 occurs upon pro-hypertrophic stimulation by the PKC-coupled agonists, PE, ANGII or endothelin I (ET-I). This pathological activated complex has been termed the hypertrophic transport metabolon (HTM) [34].

Accumulating evidence suggests a significant role of AE3 in cardiac function. AE3 Cl^−^/HCO_3_^−^ exchange activity is involved in cardiac contractility by altering cardiac Ca^++^ handling [42]. Moreover, disruption of the *ae3* gene in mice resulted in an exacerbated cardiac function and precipitated heart failure in hypertrophic cardiomyopathy mice [43]. Pacing of AE3 null hearts abrogated frequency-dependent inotropy, which, suggests that AE3 is required in mediating force-frequency response induced by acute biochemical stress [44]. Taken together, these findings suggest that the AE3 Cl^−^/HCO_3_^−^ exchanger is critical in heart growth and function but the exact mechanism remains unknown.

In the present study, we examined the role of AE3 in cardiomyocyte hypertrophy, using AE3-deficient (*ae3*^*−/−*^) mice. Cardiac growth parameters and fetal gene reactivation were measured in the presence of pro-hypertrophic stimulation in cardiomyocytes from *ae3*^*−/−*^ mice. We also examined the role of AE3 in cardiomyocyte steady state pH_i_, using the *ae3*^*−/−*^ mice. Our results indicate that *ae3* deletion prevents cardiomyocyte hypertrophy and reduces the rate of pH_i_ recovery in cardiomyocytes, reinforcing the importance of AE3 in cardiovascular pH regulation and the development of cardiomyocyte hypertrophy.

## Methods

### Animal care and use

All procedures involving animals were performed in accordance with the guidelines established by the Canadian Council on Animal Care and the University of Alberta Animal Care and Use Committee.

### ae3 null mice

Experiments were performed using *ae3* null mice in a C57BL/6 background. The *ae3* null strain has been previously described and characterized [42]. Age-matched WT mice from separate breedings were used as controls.

#### Heart weight to body weight ratio

Mice were weighed and anesthetized with sodium pentobarbital (50 mg/kg) by intraperitoneal injection. Upon reaching surgical plane, hearts were removed after performing midsection thoracotomy and rinsed in 4°C phosphate buffer saline (PBS: 140 mM NaCl, 3 mM KCl, 6.5 mM Na_2_HPO_4_, 1.5 mM KH_2_PO_4_, pH 7.4). Ventricles were separated from atria and blood vessels, blotted dry and the ventricular weight was measured. Heart weight to body weight ratio (HW/BW) was then calculated by dividing the weight of the ventricles by the weight of the whole animal.

### Hematoxylin-eosin staining of heart sections

Hematoxylin and eosin (HE) staining was performed on longitudinal and transverse sections of wildtype and *ae3* null adult mouse heart, using previously described protocols [45,46]. Briefly, paraffin-embedded hearts were sectioned into 3 μm slices, which were trimmed and floated onto a water bath at 42°C, containing 50 mg/l of gelatin while gently stretching the cut sections to avoid wrinkles. Poly-L-lysine coated microscope slides were dipped under the meniscus of the water bath and a tissue slice was carefully mounted onto it. Sections were then air-dried for 16 h at 20°C, after which the slides were placed on edge in an oven and baked for 15 min at 60°C. Sections were deparaffinized by successively immersing them for 5 min with agitation in xylene, 100% ethanol and 70% ethanol, and rehydrated in Tris-EDTA buffer (1 mM EDTA, 0.05% Tween 20, 10 mM Tris, pH 9.0) for 1 min. Slides were rinsed in distilled water for 1 min with agitation. Slides were agitated for 30 s in Mayer’s hematoxylin solution (1.0 g/l hematoxylin (Sigma), sodium iodate (0.2 g/l), aluminum ammonium sulfate · 12 H_2_O (50 g/l), chloral hydrate (50 g/l) and citric acid (1 g/l) and rinsed in water for 1 min. Slides were stained in 1% eosin Y solution (1% eosin Y aqueous solution, Fisher) for 30 s with agitation. Sections were dehydrated by successively immersing it twice in 95% ethanol and twice in 100% ethanol for 30 s each. Ethanol was extracted twice in xylene, followed by addition of two drops of mounting medium (Canada Balsam, Sigma), after which the sections were covered with a coverslip. Images of transverse and longitudinal sections were collected, using a Nikon digital camera (DXM 200) mounted on top of a Nikon Eclipse E600 microscope.

### Echocardiography

Echocardiographic assessment of cardiac performance in male *WT* and *ae3*^*−/−*^ mice was performed by the Cardiovascular Research Centre Core Facility (University of Alberta). Mice were subjected to mild anesthesia by isoflurane inhalation and echocardiography parameters were measured using a Vevo 770 High-Resolution Imaging System with a 30-MHz transducer (RMV-707B; Visual Sonics, Toronto). M-mode images and a four chamber view allowed for the calculation of wall measurements, ejection fraction, fractional shortening, and mitral velocities (E and A). Mitral valve tissue motion (E’) was measured, by tissue Doppler echocardiography of the mitral septal annulus.

### Blood pressure measurements

Non-invasive blood pressure measurements were performed by the Cardiovascular Research Centre Core Facility (University of Alberta). Mice were comfortably restrained in a 26°C warming chamber (IITC Life Science) for ~15 min prior to taking blood-pressure (BP) measurements. Tail-cuff sensors were secured on the tail to occlude the blood flow, and connected to the recording device. Systolic and diastolic pressure, heart rate, blood volume and flow were obtained, using CODA6 software (Kent Scientific Corporation, Connecticut, USA).

### Isolation and culture of adult mouse cardiomyocytes

Cardiomyocytes from adult male mouse hearts were isolated and cultured with modifications of published protocols [21,47]. Briefly, adult mice were euthanized with sodium pentobarbital (50 mg/kg body mass) by intraperitoneal injection. Upon reaching surgical plane, midsection thoracotomy was performed and hearts were quickly excised and placed in 4°C perfusion solution, containing in mM: 120 NaCl, 5.4 KCl, 1.2 MgSO_4_, 5.6 glucose, 10 2,3-butanedione monoxime (BDM) (Sigma), 5 taurine (Sigma), 1.2 NaH_2_PO_4_, 10 HEPES, pH 7.4. Extra-cardiac tissues were removed and hearts were subjected to retrograde perfusion with perfusion solution to remove excess blood. Perfusion was switched for 15 min to perfusion solution at 37°C, supplemented with 0.5 mg/ml collagenase type B (Roche), 0.5 mg/ml collagenase type D (Roche), 0.02 mg/ml protease XIV (Roche) and 50 μM CaCl_2_. Ventricles, partially digested at this stage, were removed, cut into several pieces, and digested further in the same enzyme digestion solution by gentle trituration with a transfer pipette. Once the ventricles were completely digested, enzymatic digestion was terminated by addition of Digestion stop buffer I (perfusion solution, containing 10% (v/v) fetal bovine serum (FBS) (GIBCO) and 50 μM CaCl_2_). Lysates settled under gravity for 10 min at room temperature and pellets were resuspended in myocyte stopping buffer II (perfusion solution, containing 5% (v/v) FBS and 50 μM CaCl_2_). Samples were transferred to 60 mm tissue culture dish and calcium levels were increased by addition of CaCl_2_ to obtain final concentrations of 62, 112, 212, 500 and 1000 μM, sequentially at 4 min intervals at 20°C. Cells were transferred to a 14 ml culture tube and allowed to sediment for 10 min under gravity. Cells were resuspended in myocyte culture medium (Dulbecco’s Modified Eagle’s Medium/Nutrient Mixture F12-Ham (Sigma), supplemented with 10 mM BDM, 5% (v/v) FBS, 1% penicillin (GIBCO), 10 mM BDM, and 2 mM L-glutamine (GIBCO)). Myocytes were plated at a density of (0.5-1) x 10^4^ cells/cm^2^ onto 35 mm culture dishes, pre-coated for 2 h with 10 μg/ml mouse laminin (Invitrogen) in PBS. Cells were incubated at 37°C in a 5% CO_2_ incubator for 1 h at which point medium was replaced with Dulbecco’s Modified Eagle’s Medium/Nutrient Mixture F12-Ham, containing 10 mM BDM, 1% penicillin, 2 mM L-glutamine, 0.1 mg/ml bovine serum albumin, and 1x ITS Liquid Media Supplement (Sigma). The entire culture procedure was carried out in a sterile laminar flow hood.

### Assessment of cardiomyocyte hypertrophic growth

Cardiomyocytes were isolated and cultured from adult mouse hearts as described above. Following 18 h of culture, myocytes were treated with solvent carrier (control), 10 μM phenylephrine (Sigma) or 1 μM angiotensin II (Sigma) for another 24 h. Hypertrophy was assessed by analysis of cell surface area of cardiomyocytes pre- and post-treatment with the hypertrophic agonists. Images of characteristic rod-shaped cardiomyocytes were collected with a QICAM fast-cooled 12-bit colour camera (QImaging Corporation). Cell surface areas were measured, using Image-Pro Plus software (Media Cybernetics). Each treatment group contained 100–200 cells of ~ ten different experiments. Cell surface area (% relative to control) = Surface area (post-treatment)/Surface area (pre-treatment) X 100.

### Real-time quantitative reverse transcription PCR (qRT-PCR)

Cardiomyocytes were prepared and treated as above. At the end of the culture period, medium was aspirated and cardiomyocytes were harvested in 350 μl Buffer RLT (Qiagen). RNA was extracted from the lysates with an RNeasy Plus Mini Kit, as per manufacturer’s instructions (Qiagen). RNA samples (100 ng) were reverse transcribed, following the manufacturer’s instructions for SuperScript II™ reverse transcriptase (Invitrogen). qRT-PCR was performed in a Rotorgene 3000 real time thermal cycler (Corbett Research), using a reaction mix containing: 5 μl template cDNA, 12 μl 2x Rotor-Gene SYBR Green PCR Master Mix (Rotor-Gene SYBR Green PCR Kit, Qiagen), and 1 μM of each primer. Data were obtained and analyzed, using Rotor Gene 6.0.14 software. Cycle threshold (Ct) values were obtained for carbonic anhydrase II (CAII), NHE1, atrial natriuretic peptide (ANP), β-MHC and glyceraldehyde-3-phosphate dehydrogenase (GAPDH). Primers (Table 1) were designed, using Primer3 (http://Frodo.wi.mit.edu/primer3/).

**Table 1:** Sequences of primers used in qRT-PCR

**Target gene**	**Primer sequence**	
*Anp*	F:	5′-TCCAGGCCATATTGGAGCAAATCC-3′
	R:	5′-TCCAGGTGGTCTAGCAGGTTCTTG-3′
*β-mhc*	F:	5′-GAGACGGAGAATGGCAAGAC-3′
	R:	5′-AAGCGTAGCGCTCCTTGAG-3′
*Caii*	F:	5′-CTCTGCTGGAATGTGTGACCT-3′
	R:	5′-GCGTACGGAAATGAGACATCTGC-3′
*Nhe1*	F:	5′-TTTTCACCGTCTTTGTGCAG-3′
	R:	5′-TGTGTGGATCTCCTCGTTGA-3′
*Gapdh*	F:	5′-CCTCGTCCCGTAGACAAAAT-3′
	R:	5′-TGATGGCAACAATCTCCACT-3′

*Anp*, atrial natriuretic peptide; *β-mhc* , beta myosin heavy chain*; Caii*, carbonic anhydrase II; F, Forward; *Nhe1*, sodium proton exchanger isoform 1; *Gapdh*, glyceraldehyde 3-phosphate dehydrogenase; R, Reverse.

### Immunoblotting

Cardiomyocytes, isolated and cultured from adult mouse hearts, were subjected to drug intervention as described above. Twenty-four h following treatment, medium was aspirated and myocytes were washed with 4°C PBS. Cells were lysed with SDS-PAGE sample buffer (10% (v/v) glycerol, 2% (w/v) SDS, 2% 2-mercaptoethanol, 0.001% (w/v) bromophenol blue, 65 mM Tris, protease inhibitors (1 μg/ml) pH 6.8) and lysates were heated 5 min at 65°C. Protein concentrations were determined by the bicinchoninic acid (Pierce Biotechnology) assay [48], and 20 μg of protein was resolved by SDS-PAGE on 10% acrylamide gels. Proteins were transferred onto PVDF membranes by electrophoresis for 1 h at 100 V in transfer buffer (10% (v/v) methanol, 25 mM Tris, and 192 mM glycine). PVDF membranes were blocked for 30 min with 5% (w/v) nonfat dry milk/ 0.1% (v/v) Tween 20 in TBS (137 mM NaCl, 20 mM Tris, pH 7.5). Immunoblots were incubated with rabbit polyclonal anti-CAII antibody (Santa Cruz Biotechnology; 1:1000), rabbit anti-human SLC26a6 (1:1000) [49], or rabbit polyclonal anti-NHE1 antibody (1:1000) [32] in TBST-M for 16 h at 4°C. Immunoblots were washed with TBST (TBS, containing 0.1% (v/v) Tween 20) and incubated with donkey anti-rabbit IgG conjugated to horseradish peroxidase (Santa Cruz Biotechnology; 1:2000) or mouse anti-goat IgG conjugated to horseradish peroxidase (Santa Cruz Biotechnology; 1:2000) for 1 h at room temperature. Immunoblots were washed in TBST and visualized, using ECL reagents (Perkin Elmer) and a Kodak Imaging Station 440CF (Kodak, Rochester, NY). Proteins were quantified by densitometry, using Kodak Molecular Imaging software (version 4.0.3; Kodak). Immunoblots were stripped by incubating in 10 ml of stripping buffer (2% (w/v) SDS, 10 mM 2-mercaptoethanol, 62.5 mM Tris, pH 6.8) at 50°C for 10 min with occasional shaking, followed by three washes with TBST. Membranes were incubated with mouse monoclonal anti-β-actin antibody (Santa Cruz Biotechnology; 1:2000) for 1 h at 20°C, washed with TBST, and incubated with sheep anti-mouse IgG conjugated to horseradish peroxidase (Santa Cruz Biotechnology; 1:3000) for 1 h. Immunoblots were washed and visualized again, as described above.

### Protein synthesis assays

Cardiomyocytes were prepared and subjected to hypertrophic stimulation as above. Radiolabeled phenylalanine ([^3^H]-Phe, 1 μCi/ml, (Perkin Elmer)) was added immediately after drug intervention and cells were incubated for another 24 h. Proteins were precipitated, using trichloroacetic acid (TCA) as described previously with some modifications [50]. Medium was carefully aspirated and 500 μl of 0.5% (v/v) Triton X-100, containing protease inhibitor cocktail (Roche) were added. Lysates were transferred into 1.5 ml microcentrifuge tubes and TCA (100%: 500 g in 350 ml H_2_O) was added to each tube to a final concentration of 40% (v/v). Samples were incubated at 4°C for 30 min, after which proteins were sedimented by centrifugation at 12 682 x g for 15 min at 4°C. Pellets were resuspended by adding 200 μl acetone (−20°C) and sedimented again by centrifugation, as above, for 10 min. The acetone wash was repeated one more time, and the pellets air dried for 20 min at room temperature. Pellets were resuspended in 200 μl of 0.2 M NaOH, 1% (w/v) SDS. Scintillation fluid (Perkin Elmer, 3.5 ml) was added to each sample and the radioactivity of [^3^H]-Phe was counted in a Beckman LS6500 liquid scintillation counter.

### Measurement of pH_i_ in adult mouse cardiomyocytes

The protocol was as described previously with minor modifications [51,52]. Briefly, cardiomyocytes were isolated as described above and cultured on laminin-coated glass coverslips. Approximately 2 h later, cells were loaded with 2 μM BCECF-AM (Sigma-Aldrich, Canada) for 30 min at 37°C. Coverslips were placed in an Attofluor cell chamber (Invitrogen, Canada), then transferred onto the stage of a Leica DMIRB microscope. Perfusion with HCO_3_^−^ Ringer’s buffer solution (in mM: 128.3 NaCl, 4.7 KCl, 1.35 CaCl_2_, 20.23 NaHCO_3_, 1.05 MgSO_4_ and 11 glucose, pH 7.4) was initiated at 3.5 ml/min. Solutions were bubbled with 5% CO_2_-balanced air. Intracellular alkalosis was induced by switching to a HCO_3_^−^ Ringer’s solution, containing, 20 mM trimethylamine (TMA) (Sigma) and perfusion was continued for 3 min. Perfusion was switched back to the HCO_3_^−^ Ringer’s buffer solution. pH_i_ of individual cardiomyocytes was measured by photometry at excitation wavelengths of 502.5 nm and 440 nm with a Photon Technologies International (PTI, Lawrenceville, NJ, USA) Deltascan monochromator. Emission wavelength, 528.7 nm, was selected, using a dichroic mirror and narrow range filter (Chroma Technology Corp., Rockingham, VT, USA) and was measured with a PTI D104 photometer. At the end of experiments, pH_i_ was clamped by the high K^+^/Nigericin technique [53] in calibration solutions containing, 140 mM KCl, 1 mM MgCl_2_, 2 mM EGTA, 11 mM glucose, 20 mM BDM, 10 mM HEPES. Three pH standards spanned a range of 6.5-7.5. Steady-state pH_i_ was measured from the pH_i_ value prior to induction of alkalosis. Rate of pH_i_ recovery was measured by linear regression from first min of recovery from imposed alkalosis.

### Statistical analysis

Data are expressed as mean ± S.E.M. Statistical analyses were performed using paired *t*-tests or ANOVA where appropriate. P < 0.05 was considered significant.

## Results

### Cardiac development in *ae3*^*−/−*^ mice

**Figure 1:**
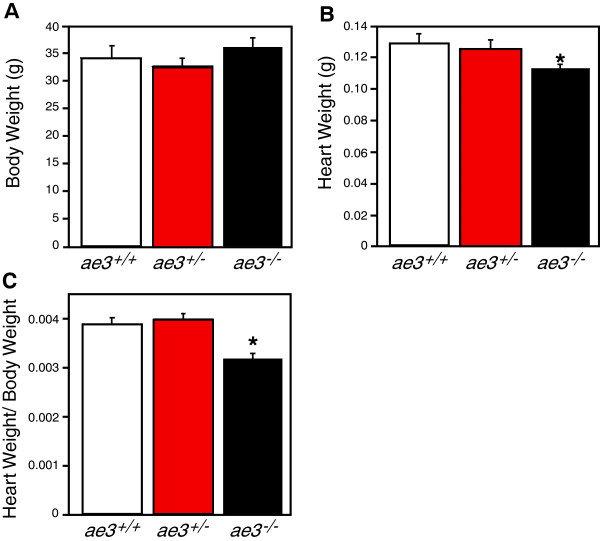
**Heart weight to body weight ratio of mice. A**, Body weights (BW) of the AE3 null (*ae3*^*-/-*^, black bar), heterozygous (*ae3*^*+/-*^, red bar) and the WT (*ae3*^*+/+*^, open bar) littermates were measured. **B**, Hearts, surgically removed from anaesthetized mice and trimmed of extra-cardiac and atrial tissue, were measured to obtain the ventricular weight (heart weight, HW). **C**, HW/BW, an index of hypertrophy, was calculated. *P<0.05 (n=8 mice/ group).

**Figure 2:**
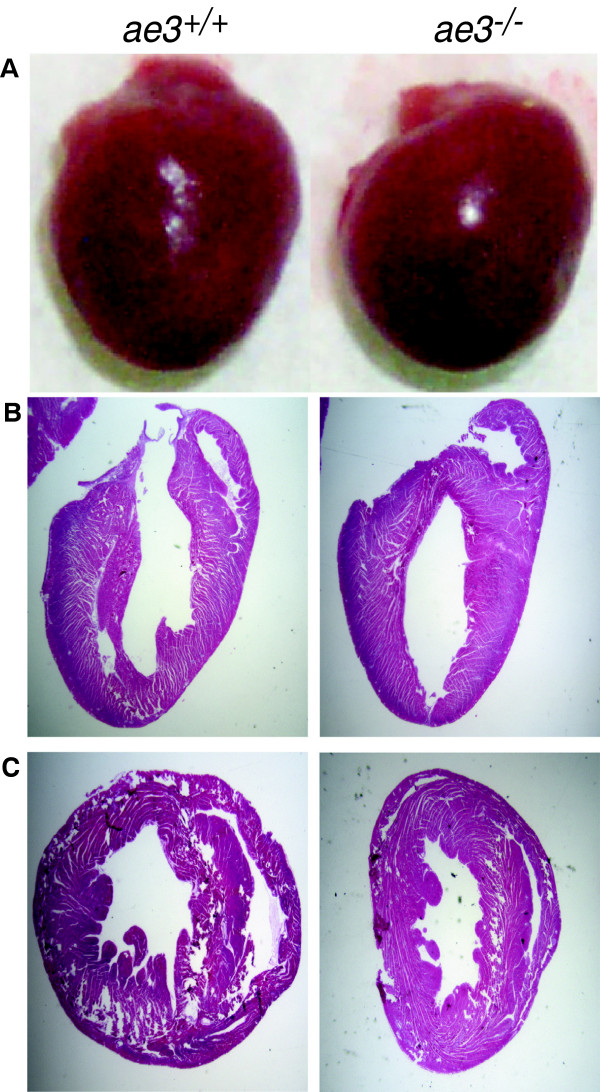
**Cross-sections of heart from WT and *ae3***^***−/− ***^**mice.** Whole hearts were removed from euthanized mice and atrial tissue was excised **(A)**. Longitudinal **(B)** and transverse **(C)** sections of the ventricle from *ae3*^*−/−*^ (Right Panel) and *ae3*^*+/+*^ (Left Panel) hearts were stained with hematoxylin/eosin.

To examine the role of AE3 in heart development, we characterized age-matched wildtype (WT) and *ae3*^*−/−*^ mice (~three months old). Breeding yielded Mendelian ratios of litters of both WT and *ae3*^*−/−*^ mice, indicating that loss of the AE3 does not affect prenatal survival. While the body weights of age-matched WT and *ae3*^*−/−*^ mice were not significantly different (Figure 1A), the difference in heart weights between the groups was statistically significant (Figure 1B). As a result, the HW/BW ratio, an index of cardiac hypertrophy, was reduced in the *ae3*^*−/−*^ mice relative to their WT counterparts (Figure 1C and D), suggesting that AE3 has a role in heart growth. Despite the observed apparent smaller size of the *ae3* null hearts, gross morphology of hearts stained with hematoxylin/eosin transverse and longitudinal sections revealed no differences in the wall size and the chamber diameter of WT (left panel) and *ae3* null (right panel) mouse hearts (Figure 2B-C).

### Blood pressure and echocardiography

Cardiovascular performance of age-matched *WT* and *ae3*^*−/−*^ mice was assessed by echocardiography. No major variations in cardiovascular functional parameters between WT and *ae3*^*−/−*^ mice (~three months old males) were observed, except for a significant decrease in the mitral value E/A ratio in *ae3*^*−/−*^ mice (Table 2). While this would suggest that more blood is entering the ventricle during the atrial systolic phase than during ventricular relaxation, other parameters of diastolic cardiac function (E/E’ ratio, IVRT) were unaffected. *ae3*^*−/−*^*mice therefore* likely do not exhibit diastolic dysfunction. Systemic blood pressure measurements were also performed using the non-evasive tail cuffing approach. No significant difference in the systemic blood pressure of *WT* and *ae3*^*−/−*^ mice was observed (Table 2). Overall, these observations suggest that loss of AE3 does not affect cardiovascular performance under basal conditions, consistent with previous findings [42,44].

**Table 2:** **Echocardiographic and blood pressure analysis of *WT *** **and *ae3***^***−/− ***^**mice**

**Parameter**	***WT***	***ae3***^***−/−***^
Ejection fraction, %	65 ± 3	67 ± 7
Fractional shortening, %	35 ± 3	37 ± 7
IVSd, mm	0.70 ± 0.01	0.75 ± 0.04
LVIDd, mm	3.5 ± 0.1	3.4 ± 0.1
LVPWd, mm	0.67 ± 0.03	0.70 ± 0.03
IVSs, mm	1.08 ± 0.03	1.11 ± 0.09
LVIDs, mm	2.3 ± 0.1	2.1 ± 0.3
LVPWs, mm	1.05 ± 0.07	1.05 ± 0.12
MV E Velocity, mm.s^−1^	730 ± 50	710 ± 50
MV A Velocity, mm.s^−1^	390 ± 30	520 ± 40
MV E/A ratio	1.9 ± 0.1	1.4 ± 0.1
E/E’	24 ± 3	21 ± 4
IVRT, ms	16 ± 0	17 ± 2
TEI index	0.63 ± 0.03	0.61 ± 0.02
Systolic pressure, mm Hg	102 ± 7	100 ± 9
Diastolic pressure, mm Hg	66 ± 6	64 ± 8
Flow, ml.min^−1^	11 ± 1	10 ± 1
Volume of blood, ml	36 ± 6	31 ± 5
Heart rate, beats.min^−1^	699 ± 1	721 ± 49

All experiments were performed on male mice. Values are expressed as means ± S.E.M. (n = 3 per group for echocardiography; n = 7 for tail cuff parameters, with three replicates over three separate days (bottom five rows of the table)); P < 0.05. IVSd, diastolic intraventricular septal wall thickness; LVIDd, diastolic left ventricular internal diameter; LVPWd, diastolic left ventricular posterior wall thickness; IVSs, systolic intraventricular septal wall thickness; LVIDs, systolic left ventricular internal diameter; LVPWs, systolic left ventricular posterior wall thickness; MV E velocity, peak E wave mitral valve velocity; MV A velocity, peak A wave mitral valve velocity; MV E/A, ratio of peak E wave mitral valve velocity to peak A wave mitral valve velocity; E/E’, ratio of peak E wave mitral valve velocity to E wave mitral valve tissue motion; IVRT, isovolumic relaxation time.

### Cardiomyocyte growth upon pro-hypertrophic stimulation

**Figure 3:**
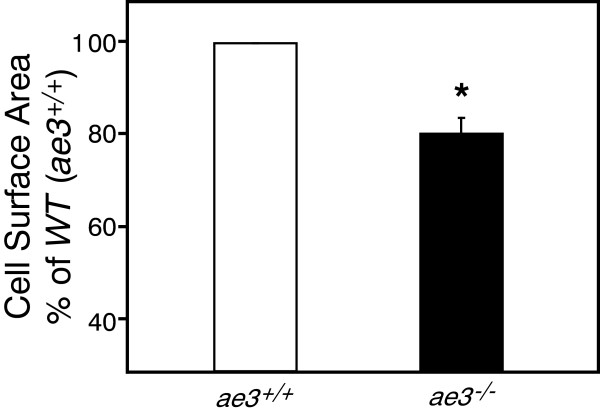
**Cardiomyocyte size in WT and *ae3***^***−/−***^**mice.** Cardiomyocytes were isolated from male adult hearts of wildtype (*ae3*^*+/+*^, open bar) and knock-out (*ae3*^*−/−*^, black bar) mice. Total cell surface area of rod-shaped cardiac cells was determined by morphometry. Data are expressed as a percentage relative to the wildtype cells. * P < 0.05 compared to WT (n = 6 mice in each group).

**Figure 4:**
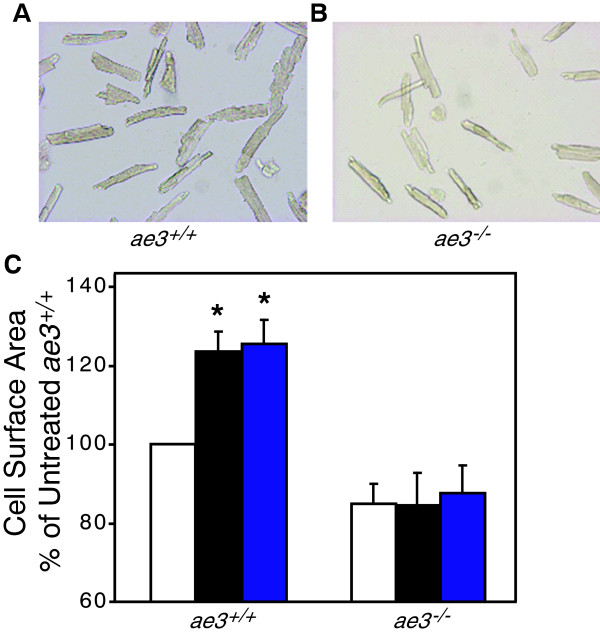
**Effect of hypertrophic stimuli on cardiomyocyte size.** Cardiomyocytes isolated from the ventricles of WT (*ae3*^*+/+*^*)***(A)** and *ae3*^*−/−*^**(B)** adult mice were subjected to vehicle alone (control), ANGII (1 μM) and PE (10 μM) treatment for 24 h, following an 18 h pre-treatment period. Images of the cardiomyocytes, taken pre- and post-treatment using a QICAM fast cooled 12-bit color camera, were quantified to measure the cell surface area. In the control group, equal volume of the vehicle was added. **C**, Cell surface areas were expressed as a percentage of vehicle-treated control groups (open bars) and compared to the ANGII (black bars) and PE (blue bars) treated groups. *P < 0.05, relative to control group (n = 10 mice in each group).

Cardiomyocyte hypertrophy is characterized by an increase in cardiomyocyte surface area, resulting in an overall increase in heart size. Cardiomyocytes were isolated from adult WT and *ae3*^*−/−*^ mice and the cell surface assessed by morphometry. The cell surface area of *ae3* null cardiomyocytes was 20% ± 4% (n = 6) lower than WT (Figure 3). To determine the response of cardiomyocytes to pro-hypertrophic stimulation, adult cardiomyocytes were cultured and treated with PE and ANGII 18 h later. Cell surface area was measured 24 h following treatment with hypertrophic agonists. PE and ANGII induced a 20–25 ± 4% (n = 10) increase in the cell surface area of WT cardiomyocytes, but cardiomyocytes from *ae3*^*−/−*^ hearts were not susceptible to pro-hypertrophic stimulation by these agents (Figure 4). This suggests that AE3 has a role in the hypertrophic signaling pathway downstream of PE and ANGII.

### Expression of hypertrophic marker genes

**Figure 5:**
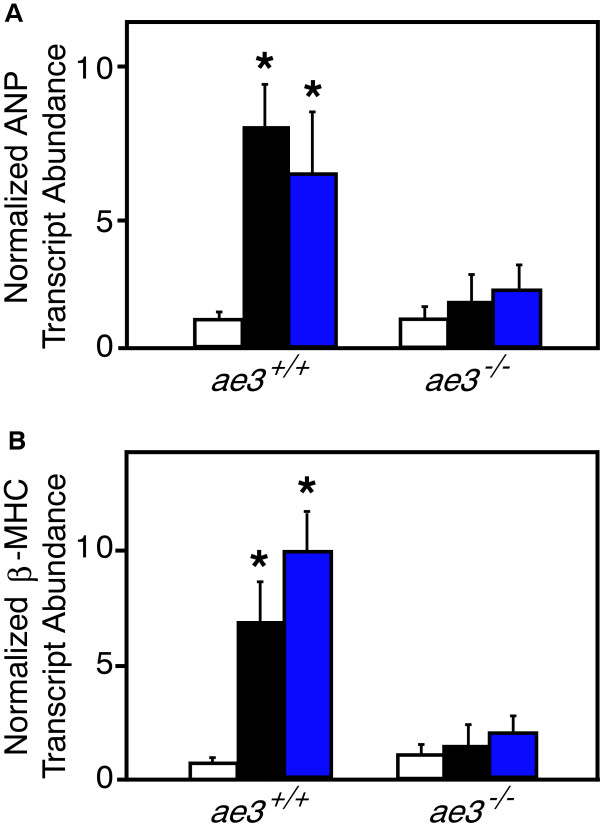
**Effect of hypertrophic stimuli on expression of hypertrophic marker mRNA.** Cardiomyocytes were isolated from *ae3*^*−/−*^ and *ae3*^*+/+*^ adult mouse hearts and maintained in cell culture. Following 18 h culture period, vehicle alone (control, open bars), ANGII (1 μM, black bars) or PE (10 μM, blue bars) were added for further 24 h. To determine the mRNA expression levels of ANP or β-MHC, quantitative real-time PCR was performed. RNA prepared from cardiomyocytes was reverse transcribed and the resulting cDNA was subjected to qRT-PCR. Cycle threshold (Ct) value was obtained for ANP or β-MHC and GAPDH. Ct values of each sample were corrected for the respective GAPDH Ct values. Absolute differences in gene expression between samples is based on the relation that a difference of one cycle corresponds to a difference of two-fold in template abundance. Relative transcript abundance was expressed as fold-change of ANP **(A)** or β-MHC **(B)** relative to control. *P < 0.05, compared to control group (n = 4).

Cardiac hypertrophic development is associated with increased expression of marker genes, including ANP [54], β-myosin heavy chain (β-MHC) [55] and α-skeletal actin [56]. mRNA and protein levels of these markers are elevated in hypertrophic hearts [57]. Expression levels of ANP and β-MHC, were assessed by qRT-PCR in cardiomyocytes subjected to pro-hypertrophic stimulation. Transcript abundance of ANP and β-MHC were not significantly different between untreated cardiomyocytes from WT and *ae3*^*−/−*^ mice (Figure 5A and B). Stimulation with PE and ANGII, however, led to 8–10 fold increase of ANP and β-MHC expression levels in cardiomyocytes from WT mice. In contrast, ANP and β-MHC increased only 1–2 fold in cardiomyocytes from *ae3*^*−/−*^ mice. These data suggest that pro-hypertrophic stimulation induces a much greater upregulation of hypertrophic marker genes in cardiomyocytes from WT mice than *ae3*^*−/−*^ cardiomyocytes.

### Expression of HTM genes in mouse cardiomyocytes

**Figure 6:**
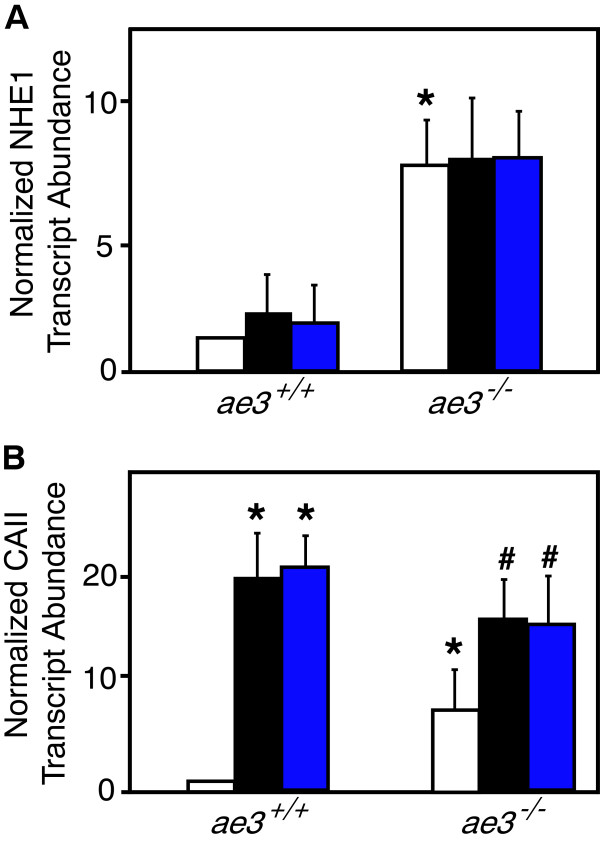
**Effect of hypertrophic stimuli on expression of CAII and NHE1 mRNA.** Cardiomyocytes were isolated from *ae3*^*−/−*^ and *ae3*^*+/+*^ adult mouse hearts. Following 18 h culture period, vehicle (open bar, control), ANGII (1 μM, black bars) or PE (10 μM, blue bars) were added for further 24 h. To determine the mRNA expression levels of NHE1 or CAII, quantitative real-time PCR was performed. RNA prepared from cardiomyocytes was reverse transcribed and the resulting cDNA was subjected to qRT-PCR. Cycle threshold (Ct) value was obtained for NHE1 or CAII and GAPDH. Ct values of each sample were corrected for the respective GAPDH Ct values. Absolute differences in gene expression between samples is based on the relation that a difference of one cycle corresponds to a difference of two-fold in template abundance. Relative transcript abundance was expressed as fold-change of NHE1 **(A)** or CAII **(B)** relative to control. *P < 0.05, compared to *ae3*^*+/+*^ control group. # P < 0.05, compared to *ae3*^*−/−*^ control group (n = 4).

AE3 is implicated as part of a functional complex with NHE1 and CAII, referred to as the hypertrophic transport metabolon (HTM), whose activation has been proposed to induce cardiac hypertrophy [32,33]. To assess the possibility of functional compensation for loss of AE3 by altered expression of these partner proteins, we examined mRNA expression of NHE1 and CAII by qRT-PCR. Baseline expression level of NHE1 in *ae3*^*−/−*^ mice was significantly elevated compared to the *WT* mice (Figure 6A). Pro-hypertrophic stimulation with PE or ANGII did not influence the NHE1 transcript abundance in the *WT* or *ae3*^*−/−*^ cardiomyocytes (Figure 6A). CAII transcript abundance, however, was higher in cardiomyocytes from *ae3*^*−/−*^*m*ice than from WT mice (Figure 6B). *ae3* ablation thus induces a compensatory increase in CAII expression. Interestingly, pro-hypertrophic stimulation markedly increased CAII transcript levels in both WT and *ae3*^*−/−*^ cardiomyocytes (Figure 6B).

Since the baseline expression level of CAII was elevated in *ae3*^*−/−*^ cardiomyocytes and was further enhanced by hypertrophic stimulants in *WT* cardiomyocytes, we evaluated CAII protein expression. Cardiomyocytes isolated from *ae3*^*−/−*^ and *WT* male adult mice hearts were probed for CAII on immunoblots (Additional file 1: Figure S1A). CAII migrated at ~27 kDa, consistent with the expected molecular weight of CAII. Both the steady-state level of CAII protein (Additional file 1: Figure S1B) and mRNA level for CAII (Figure 6B) were higher in *ae3*^*−/−*^ cardiomyocytes than *WT* cardiomyocytes. Importantly, however, there was a large quantitative difference in the response, where CAII protein level rose by about 50%, whereas CAII message rose was about eight-fold higher in the *ae3*^*−/−*^ mice than *WT*. PE and ANGII increased CAII levels in the *WT* cardiomyocytes. Contrastingly, CAII levels in *ae3*^*−/−*^ cardiomyocytes were not significantly affected by pro-hypertrophic stimulation (Additional file 1: Figure S1B).

### Protein synthesis in pro-hypertrophically-stimulated cardiomyocytes

**Figure 7:**
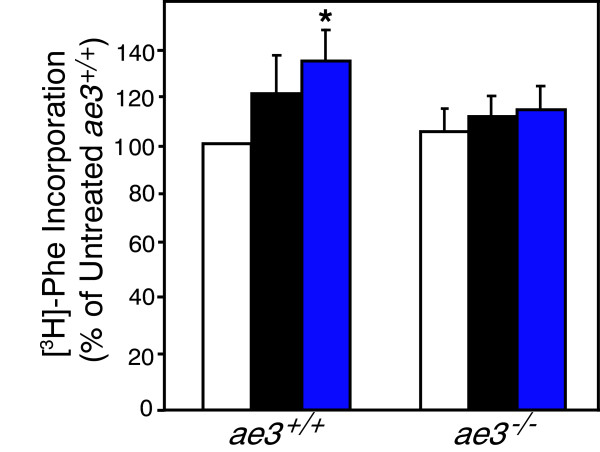
**Effect of hypertrophic stimuli on protein synthesis.** Cardiomyocytes were isolated from both *ae3*^*−/−*^ and *ae3*^*+/+*^ (wildtype) adult mice heart. Following 18 h culture period, vehicle (open bars, control), ANGII (1 μM, black bars) and PE (10 μM, blue bars) were added for further 24 h. Radiolabeled phenylalanine ([^3^H]-Phe, 1 μM), was added immediately after drug intervention and cells were incubated for further 24 h. Cells were harvested and proteins were precipitated by TCA precipitation. Incorporated [^3^H]-Phe was measured by scintillation counting and expressed relative to the vehicle control group as a percentage. *P < 0.05 compared to control group (n = 5).

Cardiac hypertrophy is also characterized by an increase in protein synthesis to accommodate cardiomyocyte enlargement [58]. Protein synthesis was measured by determining the amount of radioactive [^3^H]-Phe incorporated into proteins in cardiomyocytes, isolated and cultured as described above and treated with PE and ANGII. [^3^H]-Phe incorporation into proteins in the presence of pro-hypertrophic stimuli was increased in cardiomyocytes from WT, but not *ae3*^*−/−*^ mice. We conclude that *ae3*^*−/−*^ mice do not respond to pro-hypertrophic agonists with increased protein synthesis (Figure 7).

### pH_i_ regulation in mouse cardiomyocytes

**Figure 8:**
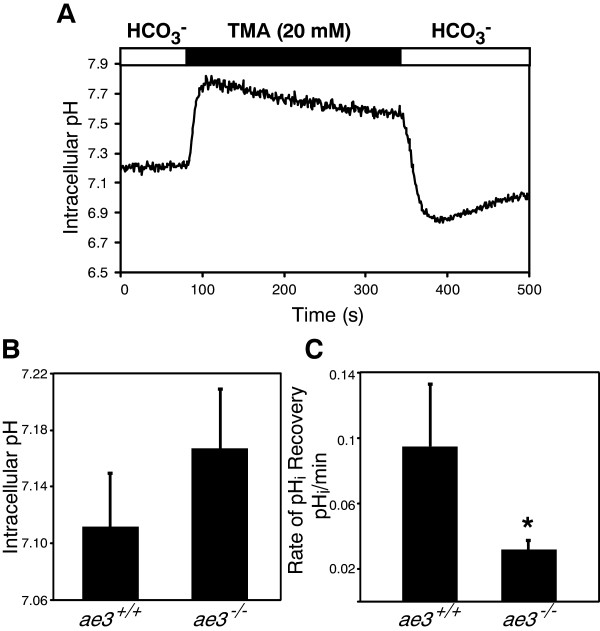
**Intracellular pH regulation in *ae3***^***−/− ***^**cardiomyocytes.** Freshly isolated cardiomyocytes were loaded with 2 μM BCECF-AM for 30 min, placed in an Attofluor cell chamber and mounted onto an inverted epifluorescence Leica DMIRB microscope. **A**, In this representative experiment, WT cardiomyocytes were perfused with HCO_3_^−^-containing Ringer’s buffer (open bar) until steady-state pH was reached and perfusion was switched to HCO_3_^−^-containing Ringer’s buffer supplemented with 20 mM TMA (black bar). Perfusion was switched to the HCO_3_^−^-containing Ringer’s buffer ~3 min later. At the end of the perfusion pH_i_ was clamped by the high K^+^/nigericin technique to convert fluorescent intensities to pH_i_ (not shown). **B**, Steady-state pH_i_ was measured as the pH_i_ value prior to induction of intracellular alkalosis. **C**, The rate of recovery of pH_i_ from imposed alkalosis was assessed as the first minute of pH_i_ recovery fitted by linear regression. *P < 0.05 compared to WT (*ae3*^*+/+*^) (n = 4).

AE3 contributes to cardiomyocyte pH_i_ regulation [59-61], but there are no AE3-specific inhibitors that would enable delineation of the role of AE3 in pH_i_ regulation. We thus examined the rate of recovery of pH_i_ from an imposed intracellular alkalinization in freshly isolated cardiomyocytes. Cardiomyocytes were cultured on laminin-coated glass coverslips for 2 h, then incubated with the pH-sensitive fluorescence dye, BCECF-AM. Cells were perfused with a HCO_3_^−^-containing Ringer’s buffer until steady-state pH_i_ was reached and the perfusion was switched to the HCO_3_^−^-containing Ringer’s buffer, containing TMA. The presence of TMA induced a rapid intracellular alkalinization (Figure 8A) until a new steady-state pH_i_ was reached. pH_i_ regulatory transporters spontaneously restored cell pH_i_. Steady-state pH_i_ was measured as the pH_i_ 60 s prior to switching to the TMA-supplemented HCO_3_^−^-containing Ringer’s buffer. The rate of pH_i_ recovery from imposed intracellular alkalinization was measured by linear regression of the initial minute of pH_i_ recovery. Baseline pH_i_ was identical in cardiomyocytes from WT and *ae3*^*−/−*^ mice (Figure 8B). The mean peak pH following alkalization for WT group was 7.65 ± 0.05 and 7.60 ± 0.06 for *ae3*^*−/−*^ mice. Thus, under basal physiological conditions, loss of AE3 does not significantly affect the steady-state pH_i_ in cardiomyocytes. The rate of recovery of pH_i_ from alkalosis was, however, significantly slower in *ae3*^*−/−*^ cardiomyocytes than WT (Figure 8C).

### Expression of pH_i_ regulators in *ae3*^*−/−*^ cardiomyocytes

We next assessed the expression level of the other pH_i_ regulatory transporters at the protein level. On immunoblots the proteins migrated at the expected sizes, NHE1 at ~100 kDa, and SLC26a6 at ~80 kDa (Additional file 2: Figure S2 and Additional file 3: Figure S3). Consistent with a previous study [44], expression of NHE1 was elevated in *ae3*^*−/−*^ cardiomyocytes compared to WT, but remained unchanged in the heterozygotes (Additional file 2: Figure S2A-B). Expression of SLC26a6 protein was not affected by deletion of the *ae3* gene (Additional file 3: Figure S3A-B).

## Discussion

Pathological cardiac hypertrophy renders the heart susceptible to cardiac failure. Accumulating evidence implicates NHE1 as a key candidate mediating pathological hypertrophy. Prolonged NHE1 activation produces intracellular alkalinization. Sustained NHE1 activation can only occur in the presence of a counter acidifying mechanism. The present study examined the possibility that the Cl^−^/HCO_3_^−^ anion exchange mediated by AE3 is responsible for the acidification mechanism. Our studies, using AE3 deficient mice, support a role for AE3 in cardiovascular pH regulation and the development of hormonally-induced cardiomyocyte hypertrophy. Pharmacological antagonism of AE3 is thus a possible therapeutic direction in the prevention of maladaptive cardiac hypertrophy.

### Role of AE3 in cardiomyocyte hypertrophy

The role of AE3 in cardiac physiology is incompletely characterized, but several lines of evidence suggest that AE3 Cl^−^/HCO_3_^−^ exchange is required to maintain pH_i_ homeostasis [10,61]. Consistent with this, we found that the rate of recovery from an alkaline load was reduced in *ae3*^*−/−*^ cardiomyocytes, relative to WT.

The hypertrophic transport metabolon is a proposed pathological pathway in which AE3, NHE1 and CAII are coordinately activated and promote hypertrophic growth [32,33]. Specifically, pro-hypertrophic agonists, including PE, ANGII and endothelin are coupled to PKC activation. NHE1 and AE3 are both activated by agonists coupled to PKC activation [59,62,63]. Co-activation of these respective alkalinizing and acidifying transporters has the net effect of loading the cell with NaCl, with no change of cytosolic pH. Elevated cytosolic Na^+^ in turn reduces the efficacy of Ca^++^-efflux by the plasma membrane Na^+^/Ca^++^ exchanger, resulting in a rise in cytosolic Ca^++^. Ca^++^ is a pro-hypertrophic second messenger [64,65] and also may promote hypertrophy in a feed-forward cascade by stimulation of PKC [6]. Accumulating evidence suggests that both NHE1 and AE3 activities are influenced by physical and functional interactions with CAII [32,41], which provides substrates for these transporters. That said, very recent data suggest that intracellular carbonic anhydrase does not activate cardiomyocyte Na^+^/H^+^ exchange [66]. Prevention of PE-induced cardiomyocyte hypertrophy upon CAII blockade [32,33] may thus arise from reduced AE3-mediated cytosolic acidification, thereby decreasing driving force for NHE-mediated alkalinization. We propose that CAII, NHE1 and AE3 form a hypertrophic transport metabolon, where hypertrophy is promoted by the pathological activation of AE3 and NHE1, stimulated by interactions with CAII.

The functional relationship between AE3, CAII and NHE1 was further supported by our analysis of protein expression in *ae3*^*−/−*^ mice. Increased CAII transcript abundance and protein expression in *ae3*^*−/−*^ mice compared to WT mice suggest that there is compensation for a loss of AE3. This finding parallels results in retinal tissue from *ae3*^*−/−*^ mice, where there was increased CAII expression [67]. Functional complementarity of AE3 and CAII is further supported by the significant increase in AE3 transcript abundance in *Car2* (*caii*^*−/−*^) mice, compared to WT mice [33]. The upregulation of NHE1 transcript abundance and increased protein expression provide further support for the HTM. Taken together, these data support a functional interaction between AE3 and CAII, where there is compensation of one for the loss of the other.

### Loss of AE3 prevents cardiomyocyte hypertrophy

This study lends support to the idea that AE3 is the Cl^−^/HCO_3_^−^ exchanger isoform working in conjunction with NHE1 to promote cardiomyocyte hypertrophy. Non-specific inhibition of Cl^−^/HCO_3_^−^ exchangers, using stilbene derivatives, prevented hypoxia-induced acidification in rat ventricular myocytes, as well as increases in intracellular Cl^−^ and Ca^++^ concentrations [68,69], suggesting a role of Cl^−^/HCO_3_^−^ exchangers in cardiac pathology. When subjected to ischemia and reperfusion, hearts isolated from *ae3*^−/−^ mice revealed no effect on cardiac performance demonstrated as contractility, ventricular developed pressure or end diastolic pressure relative to wildtype [42]. Double knock-out *ae3*/*nkcc1* (Na^+^-K^+^-2Cl^−^ co-transporter) mice, however, had elevated ischemia/reperfusion injury, which resulted in impaired cardiac contractility and overall cardiac performance [42]. These findings were attributed to impaired Ca^++^ handling in the double knock-out cardiomyocytes [42] compared to the single mutants. In a hypertrophic cardiomyopathy mouse model carrying a Glu180Gly mutation in α-tropomyosin (TM180), disruption of *ae3* did not prevent or reverse the hypertrophic phenotype [43]. The TM180/*ae3* double knockout mice had reduced cardiac function and compromised Ca^++^ regulation, which accounted for the rapid decline to heart failure [43].

Taken together, these two studies suggest that AE3 loss is not cardioprotective, which contrasts with findings of the present study, which found that loss of AE3 renders cardiomyocytes less susceptible to pro-hypertrophic stimulation. Our data showed that the marked rise in cell surface area, protein synthesis, and fetal gene reactivation observed in response to hypertrophic stimulation in cardiomyocytes from WT mice was not present in *ae3*^*−/−*^ mice. In the context of cardiomyocyte hypertrophy mediated by hormonal stimuli, our data demonstrate that ablation of AE3 affords protection against cardiomyocyte hypertrophy.

The discrepancy could arise from the model of hypertrophy and the cardiac pathology being investigated. Hypertrophic cardiomyopathy is a genetic disorder, which occurs as a result of mutations in the genes that encode cardiac contractile proteins [70]. This anomaly manifests as sudden cardiac death, arrhythmias, hypertrophy and heart failure [70]. Overall, hypertrophic cardiomyopathy results in impairment of Ca^++^ sensitivity by the myofibrils. In the present study however, we employed a model of hypertrophy induced by PE or ANGII, which involves interaction of these ligands with their cell surface receptors, GPCR [71]. The resultant intracellular response leads to increased cytosolic Ca^++^ overload, which mediates a cascade of signaling pathway involving activation of PKC, which ultimately induces cardiomyocyte hypertrophy [72].

One other possible explanation for the discrepancy between the present findings and earlier studies of *ae3*^*−/−*^ mice is the assay of cardiac hypertrophy. Earlier investigations probed whole animal physiology, whereas we studied isolated cardiomyocytes in culture. Isolated cardiomyocytes are an established model, which may be more sensitive in detecting alterations of heart cell growth than assessments of functional alterations of heart function sued in earlier studies.

Interestingly, AE3 also forms a complex with CAXIV in the myocardium [73]. In the hypertrophic myocardium of SHR rats, exacerbated AE3/CAXIV activation was proposed to elicit hypertrophic growth of the heart [73]. Thus, the etiology, signaling pathway and pathophysiology of hypertrophic cardiomyopathy are distinct from that mediated by hormonal factors. These differences could account for the disparity between the influence of AE3 on hypertrophy in the present report and that shown in previous models of cardiovascular disease [42,43]. Since hypertrophic interventions by PE and ANGII failed to induce hypertrophy in the *ae3* null cardiomyocytes in our study, the PKC-coupled hypertrophic cascade appears to require AE3. Recently, it was demonstrated that when paced, AE3 null hearts had an impaired force-dependent inotropy characterized by an elevation of protein kinase B (PKB, Akt) phosphorylation and a downregulation of AMPK activity [44]. This observation may provide additional support of the present findings that revealed that *ae3* null cardiomyocytes are less susceptible to develop hypertrophy in upon pro-hypertrophic stimulation. Akt phosphorylation is central to the signaling pathway mediated by growth factors that induce physiological hypertrophy [57]. Thus, its activation under stressful conditions may trigger a physiological growth response to counter the pathological signaling cascade stimulated the source of stress. The lack of response by the AE3 knock-out cardiomyocytes to hypertrophic stimulants seen here could arise in part from effects on Akt phosphorylation.

### Phenotype of *ae3*^*−/−*^ mice

*ae3*^*−/−*^ mice have been reported to have no apparent defects, and the results of our analysis of the cardiac function of *ae3*^*−/−*^ mice is comparable to these previous studies [42,43,74]. Combined analysis of echocardiographic measurements of ventricular wall dimensions, chamber diameter and cardiac function between the two genotypes further suggests that loss of AE3 does not affect hypertrophy or cardiovascular performance. A significant decrease in the HW/BW ratio in *ae3*^*−/−*^ mice is the result of a reduction in heart size, arising from a decrease in cardiomyocyte size. This suggests a critical role for AE3 in heart development.

### Role of AE3 in control of cardiomyocyte pH_i_

Since cardiomyocyte steady-state pH was the same in *ae3*^*−/−*^ and WT mice, loss of Cl^−^/HCO_3_^−^ exchange activity by *ae3* deletion is likely compensated for by another protein. The principal Cl^−^/HCO_3_^−^ exchanger of cardiomyocytes is SLC26a6 [23], making it the most likely acid loading transporter to compensate for loss of AE3. Since we did not see an increase in SLC26a6 expression, SLC26a6 activation may occur through post-translational mechanisms. We did note an increase of CAII expression in *ae3*^*−/−*^ mice. Since CAII increases the rate of CO_2_/HCO_3_^−^ inter-conversion, it increases the rate of observed Cl^−^/HCO_3_^−^ for SLC26a6 [75]. Thus increased CAII expression might in part compensate for loss of AE3. We also noted a much larger increase in CAII message than we saw for CAII protein. Since proteins carry out cellular functions, not mRNA, we consider the protein change to be a more reliable measure of cell response than the mRNA increase. The reason for a muted increase in CAII protein level in comparison to the mRNA rise remains unclear.

The importance of AE3 in intracellular pH regulation was, however, evident in the reduced rate of pH_i_ recovery from imposed intracellular alkalosis in cardiomyocytes from *ae3*^*−/−*^ mice compared to WT. Endothelin 1 stimulation of myocardial Cl^−^/HCO_3_^−^ exchange activity in isolated rat papillary muscle is almost totally attributable to the AE3 isoform, on the basis of inhibition by an anti-AE3 antibody [61]. This provides a possible explanation for the resistance of *ae3*^*−/−*^ mice to pro-hypertrophic stimuli; *ae3*^*−/−*^ cardiomyocytes have reduced acidifying activity to counter enhanced NHE1 activity associated with pro-hypertrophic stimulation.

## Conclusions

We explored the role of AE3 in the development of cardiomyocyte hypertrophy and cardiovascular pH regulation, using AE3 deficient mice. Cardiomyocytes from *ae3*^*−/−*^ mice were protected from increases in cell surface area, protein synthesis, and fetal gene reactivation in response to hypertrophic stimulation. Steady-state cardiomyocyte pH_i_ in *ae3*^*−/−*^ mice was comparable to WT, but slower to recover from imposed intracellular alkalosis. Our findings demonstrate that AE3 is important in hypertrophic signaling pathways activated by PE and ANGII, possibly acting through the hypertrophic transport metabolon. Pharmacologically targeting AE3 activity in the event of hypertrophy is an attractive strategy to treat heart failure patients.

## Competing interests

The authors declare that they have no competing interests.

## Authors’ contributions

DS: First author of the manuscript and completed the majority of the experimental procedures. BFB: Assisted with manuscript preparation. AQ: Collected the qRT-PCR data and generated/optimized the respective primers. BVA: Collection of HW: BW data. JRC: Conceived and supervised the experiments and assisted with manuscript preparation. All authors read and approved the final manuscript.

## Pre-publication history

The pre-publication history for this paper can be accessed here:

http://www.biomedcentral.com/1471-2261/14/89/prepub

## Supplementary Material

Additional file 1: Figure S1 Effect of hypertrophic stimulation on cardiomyocyte CAII protein expression. Cardiomyocytes isolated from wildtype (*ae3*^*+/+*^) and knock-out (*ae3*^*−/−*^) mice hearts were cultured for 18 h and subjected to vehicle-alone (CON), angiotensin II (ANGII) and phenylephrine (PE) treatment for further 24 h. Lysates prepared from cardiomyocytes were probed for CAII expression by immunoblotting. Immunoblots were stripped and probed for β-actin. A, Upper panel, representative immunoblot of lysates probed with anti-CAII antibody; lower panel, representative immunoblot stripped and re-probed with anti-β-actin antibody. B, CAII expression normalized for β-actin expression in cardiomyocytes treated with vehicle control (open bar), ANGII (black bar) and PE (blue bar), expressed as a percentage of control. Cardiomyocytes isolated from *ae3*^*+/+*^ and *ae3*^*−/−*^*mouse* hearts, as indicated. * P < 0.05, compared to control group (n = 4). Click here for file

Additional file 2: Figure S2 Expression of NHE1 protein in WT and *ae3*^*−/−*^ mouse hearts. Cardiomyocytes isolated from adult mice hearts, were lysed and probed on immunoblots for NHE1. A, Upper panel is a representative immunoblot probed with anti-NHE1 antibody of cardiomyocyte lysates prepared from wildtype (WT), *ae3* heterozygote (*ae3*^*+/−*^) and *ae3* null (*ae3*^*−/−*^) mice; lower panel, representative immunoblot stripped and reprobed with anti-β-actin antibody. B, Summary of NHE1 amount quantified by densitometry and expressed as a percentage relative to the WT group. *P < 0.05 compared to the WT group (n = 4). Click here for file

Additional file 3: Figure S3 Expression of Slc26a6 protein in WT and *ae3*^*−/−*^ mouse hearts. Cardiomyocytes isolated from adult mice hearts, were lysed and probed on immunoblots for Slc26a6. A, Upper panel is a representative immunoblot probed with anti-Slc26a6 antibody of cardiomyocyte lysates prepared from wildtype (WT), *ae3* heterozygote (*ae3*^*+/−*^) and *ae3* null (*ae3*^*−/−*^) mice; lower panel, representative immunoblot stripped and reprobed with anti-β-actin antibody. B, Summary of Slc26a6 amount quantified by densitometry and expressed as a percentage relative to the WT group. *P < 0.05 compared to the WT group (n = 4). Click here for file
