# Supplementary figures and images for: Resistance to cardiomyocyte hypertrophy in ae3 −/− mice, deficient in the AE3 Cl−/HCO3− exchanger
Source: BMC Cardiovasc Disord. 2014 Jul 21;14:89. doi: 10.1186/1471-2261-14-89 (PMC4120010; doi:10.1186/1471-2261-14-89)

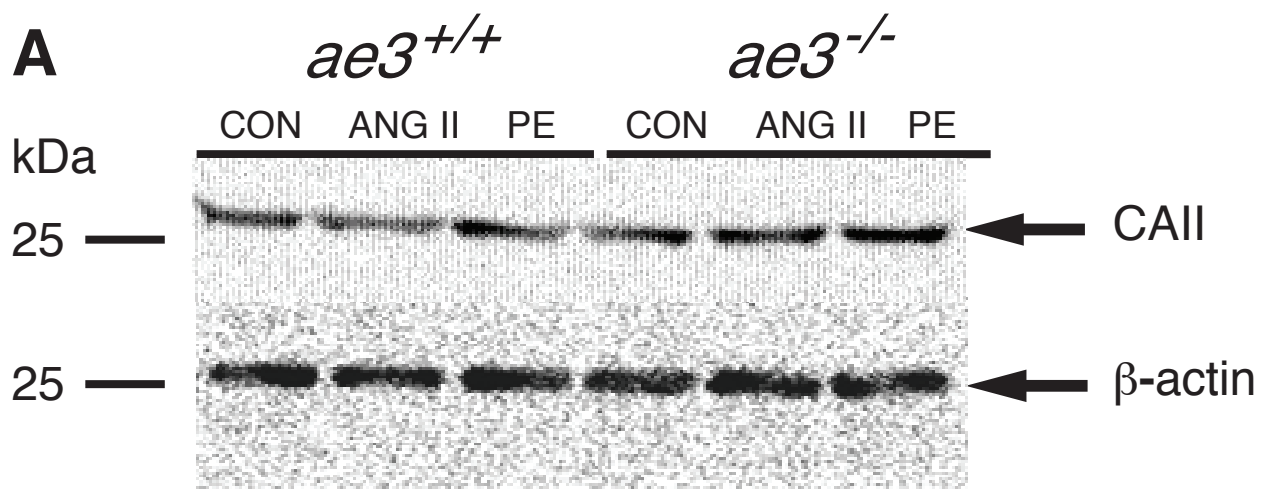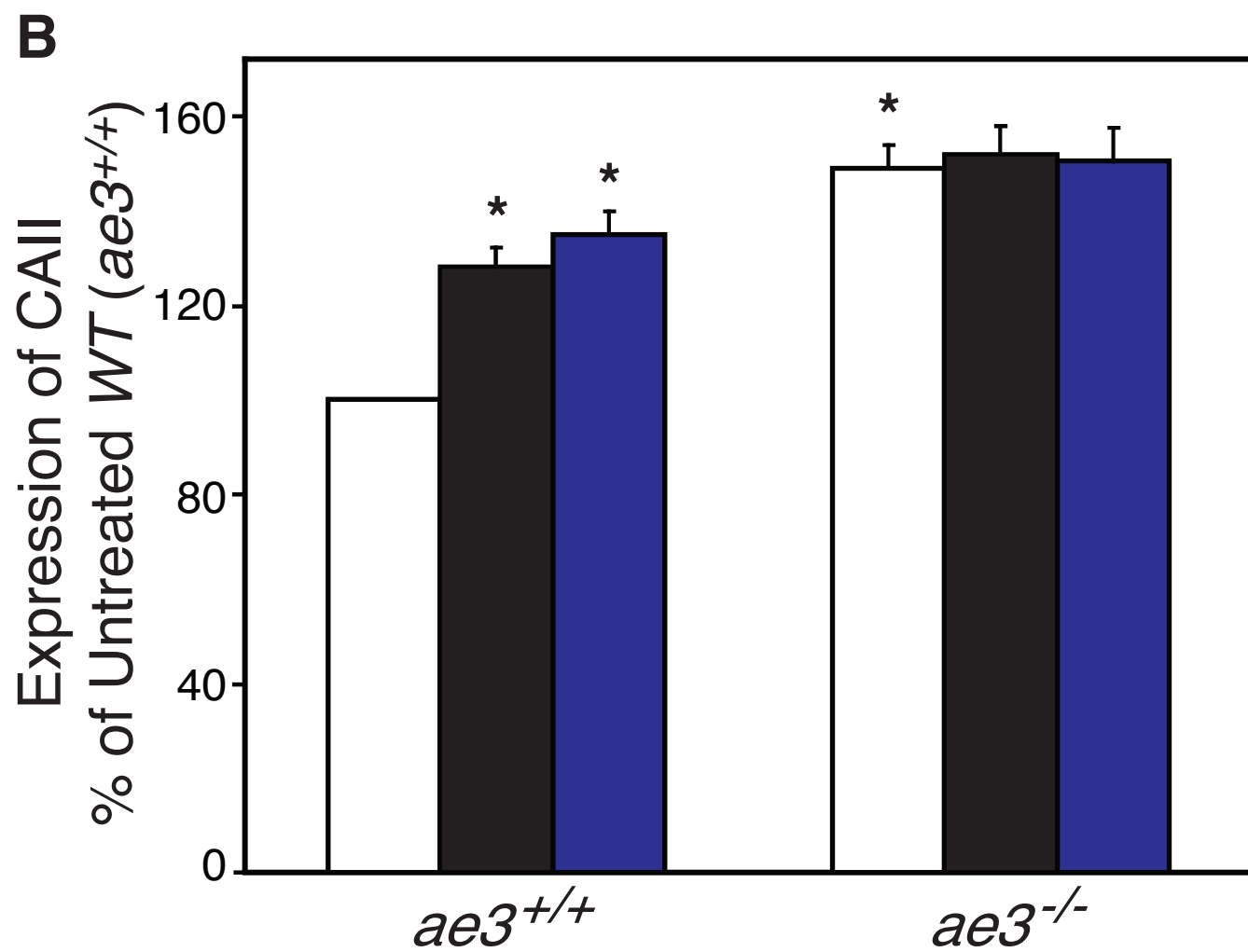

Supplementary Figure 1

Supplement: Additional file 1: Figure S1 — Effect of hypertrophic stimulation on cardiomyocyte CAII protein expression. Cardiomyocytes isolated from wildtype (ae3 +/+ ) and knock-out (ae3 −/− ) mice hearts were cultured for 18 h and subjected to vehicle-alone (CON), angiotensin II (ANGII) and phenylephrine (PE) treatment for further 24 h. Lysates prepared from cardiomyocytes were probed for CAII expression by immunoblotting. Immunoblots were stripped and probed for β-actin. A, Upper panel, representative immunoblot of lysates probed with anti-CAII antibody; lower panel, representative immunoblot stripped and re-probed with anti-β-actin antibody. B, CAII expression normalized for β-actin expression in cardiomyocytes treated with vehicle control (open bar), ANGII (black bar) and PE (blue bar), expressed as a percentage of control. Cardiomyocytes isolated from ae3 +/+ and ae3 −/− mouse hearts, as indicated. * P < 0.05, compared to control group (n = 4). [file 1471-2261-14-89-S1.pdf]

**A**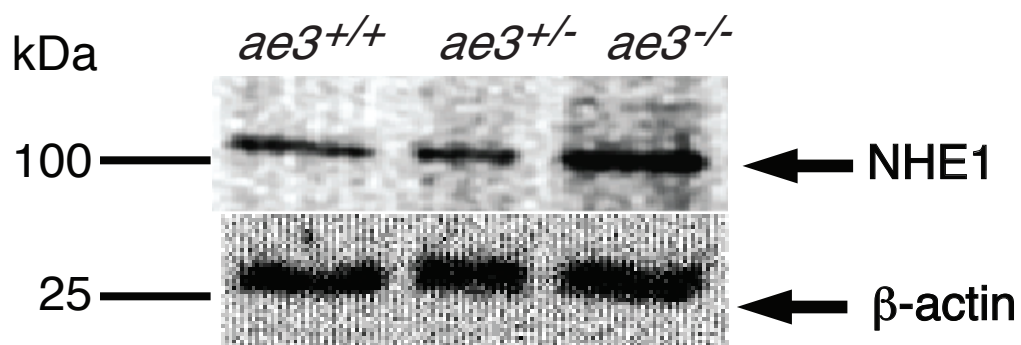**B**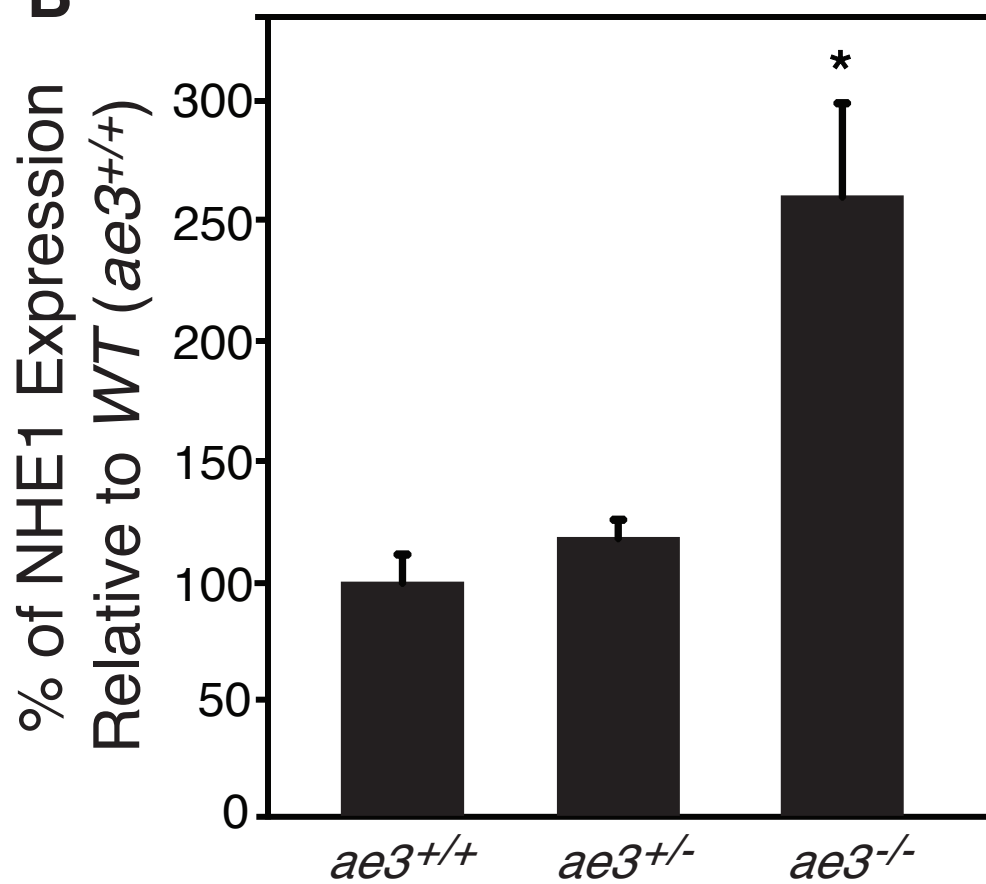

Supplementary Figure 2

Supplement: Additional file 2: Figure S2 — Expression of NHE1 protein in WT and ae3 −/− mouse hearts. Cardiomyocytes isolated from adult mice hearts, were lysed and probed on immunoblots for NHE1. A, Upper panel is a representative immunoblot probed with anti-NHE1 antibody of cardiomyocyte lysates prepared from wildtype (WT), ae3 heterozygote (ae3 +/− ) and ae3 null (ae3 −/− ) mice; lower panel, representative immunoblot stripped and reprobed with anti-β-actin antibody. B, Summary of NHE1 amount quantified by densitometry and expressed as a percentage relative to the WT group. *P < 0.05 compared to the WT group (n = 4). [file 1471-2261-14-89-S2.pdf]

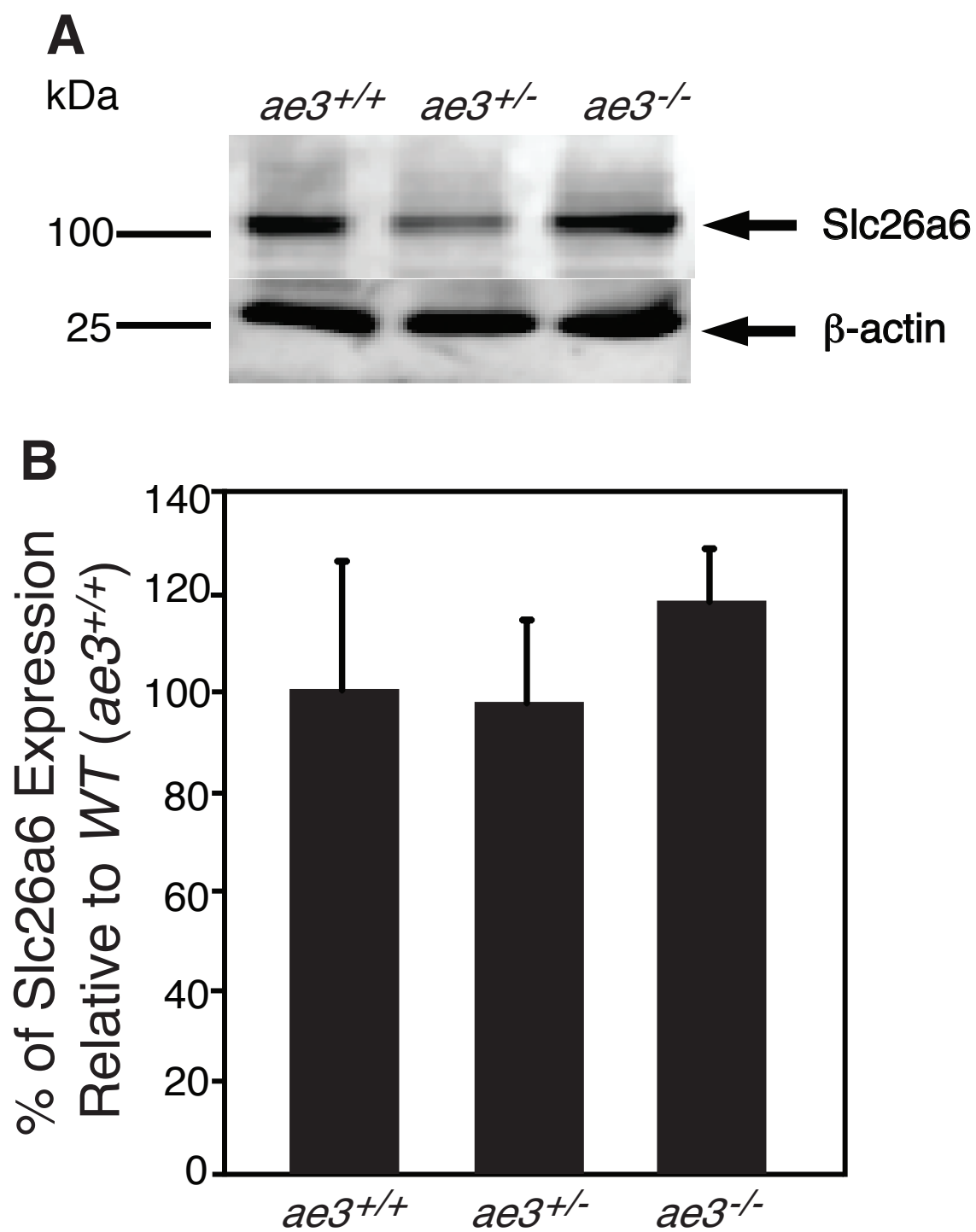

Supplementary Figure 3

Supplement: Additional file 3: Figure S3 — Expression of Slc26a6 protein in WT and ae3 −/− mouse hearts. Cardiomyocytes isolated from adult mice hearts, were lysed and probed on immunoblots for Slc26a6. A, Upper panel is a representative immunoblot probed with anti-Slc26a6 antibody of cardiomyocyte lysates prepared from wildtype (WT), ae3 heterozygote (ae3 +/− ) and ae3 null (ae3 −/− ) mice; lower panel, representative immunoblot stripped and reprobed with anti-β-actin antibody. B, Summary of Slc26a6 amount quantified by densitometry and expressed as a percentage relative to the WT group. *P < 0.05 compared to the WT group (n = 4). [file 1471-2261-14-89-S3.pdf]
